# Human umbilical cord-derived mesenchymal stromal cells for the treatment of steroid refractory grades III-IV acute graft-versus-host disease with long-term follow-up

**DOI:** 10.3389/fimmu.2024.1436653

**Published:** 2024-08-15

**Authors:** Jing-wen Niu, Yuhang Li, Chen Xu, Hongxia Sheng, Chong Tian, Hongmei Ning, Jiangwei Hu, Jianlin Chen, Botao Li, Jun Wang, Xiao Lou, Na Liu, Yongfeng Su, Yao Sun, Zhuoqing Qiao, Lei Wang, Yu Zhang, Sanchun Lan, Jing Xie, Jing Ren, Bo Peng, Shenyu Wang, Yanping Shi, Long Zhao, Yijian Zhang, Hu Chen, Bin Zhang, Liangding Hu

**Affiliations:** Senior Department of Hematology, The Fifth Medical Center of Chinese People's Liberation Army (PLA) General Hospital, Beijing, China

**Keywords:** hematopoietic stem cell transplantation, graft-versus-host disease, mesenchymal stromal cells, umbilical cord, long-term follow-up

## Abstract

**Introduction:**

Mesenchymal stromal cells (MSCs) have been extensively studied as a potential treatment for steroid refractory acute graft-versus-host disease (aGVHD). However, the majority of clinical trials have focused on bone marrow-derived MSCs.

**Methods:**

In this study, we report the outcomes of 86 patients with grade III-IV (82.6% grade IV) steroid refractory aGVHD who were treated with human umbilical cord-derived mesenchymal stromal cells (UC-MSCs). The patient cohort included 17 children and 69 adults. All patients received intravenous infusions of UC-MSCs at a dose of 1 × 106 cells per kg body weight, with a median of 4 infusions (ranging from 1 to 16).

**Results:**

The median time between the onset of aGVHD and the first infusion of UC-MSCs was 7 days (ranging from 3 to 88 days). At day 28, the overall response (OR) rate was 52.3%. Specifically, 24 patients (27.9%) achieved complete remission, while 21 (24.4%) exhibited partial remission. The estimated survival probability at 100 days was 43.7%. Following a median follow-up of 108 months (ranging from 61 to 159 months), the survival rate was approximately 11.6% (10/86). Patients who developed acute lower GI tract and liver GVHD exhibited poorer OR rates at day 28 compared to those with only acute lower GI tract GVHD (22.2% vs. 58.8%; p= 0.049). No patient experienced serious adverse events.

**Discussion:**

These finding suggest that UC-MSCs are safe and effective in both children and adults with steroid refractory aGVHD. UC-MSCs could be considered as a feasible treatment option for this challenging conditon. (NCT01754454).

## Introduction

Acute graft-versus-host disease (aGVHD) is a severe complication following allogeneic hematopoietic stem cell transplantation (HSCT), primarily affecting skin, liver and gastrointestinal (GI) tract. Despite GVHD prophylaxis, approximately 50% of transplant recipients still develop aGVHD, and 11% develop grade III to IV aGVHD (1). The prognosis for patients with grade III to IV is dismal with a 2-year survival rate of 20% and a 5-year survival rate of 8% (2). Systemic steroids remain the standard first-line treatment for acute GVHD. Approximately 40%–50% of patients develop steroid-refractory acute GVHD, which is associated with poor OS (3, 4). Currently, ruxolitinib is the only therapy approved by the European Medicines Agency (EMA) and the US Food and Drug Administration (FDA) for steroid refractory aGVHD. However, in the phase III randomized clinical trial, 38% of patients who received ruxolitinib did not achieve a CR or PR by day 28, and 60% of the patients required a third-line immunosuppressive therapy or had died by day 56 (5). An important clinical question is which treatment to use in patients with steroid refractory aGVHD who cannot afford ruxolitinib, not responding to ruxolitinib, with ruxolitinib toxicity (including cytopenias, infections), or contraindications.

In 2004, LeBlanc et al. pioneered the use of bone marrow-derived MSCs in the treatment of a pediatric patient with grade IV lower GI tract and liver aGVHD (6). This was followed by a phase II, multicenter clinical trial in 2008, which evaluated the efficacy of MSCs in steroid refractory severe aGVHD. Out of 55 patients, 39 responded favorably to MSCs treatment, without any side effects (7). Since then, MSCs have emerged as a promising therapeutic option for patients with steroid refractory aGVHD. In 2015, the Japanese Pharmaceuticals and Medical Devices Agency took the lead in granting approval to JR-031 (TEMCELL^®^) for the treatment of aGVHD in both children and adults.

Mesenchymal stem cells (MSCs) exhibit multi-lineage differentiation potential, along with nonspecific immunosuppressive and immunomodulatory effects. These cells can be isolated from various sources, including bone marrow (BM), adipose tissue, placental tissues and umbilical cord (UC) (8, 9).

Most of the large-scale analyses have focused on bone marrow-derived MSCs. However, bone marrow aspiration is an invasive procedure that may cause pain, infection or hemorrhage. And the number and function of BM-MSCs may influenced by donor age (10). Compared to BM, human umbilical cord (UC) can provide a large number of MSCs (11). It is a waste product after childbirth, and harvesting UC-MSCs does not involve invasive procedures, thus offering better ethical acceptance. What’s more, UC-MSCs have demonstrated lower immunogenicity and higher proliferative capacity (12). Despite the use of UC-MSCs to treat steroid refractory aGVHD has demonstrated promising results, the OS by day 28 ranging from 59% to 80% (13–18), the number of clinical trials is relatively few, and the history of UC-MSCs application is shorter. In this study, we report the outcomes of 86 patients with grade III-IV steroid refractory acute GVHD (all involved lower GI tract) who received UC-MSCs as a salvage therapy and with a long period of follow-up. To the best of our knowledge, this is the largest cohort of 86 grade III-IV acute GVHD patients treated with UC-MSCs reported so far.

## Subjects and methods

### Patients

Patients of all ages experiencing steroid refractory grade III to IV acute GVHD, have lower GI tract involved, were eligible for this study. The grading and staging of acute GVHD were determined using the Modified Glucksberg Criteria (19). Steroid refractory GVHD was defined as progression of acute GVHD within 3-5 days or failure to improve within 5-7 days of treatment with 2mg/kg/day of prednisone (20). A total of 86 patients were enrolled in this study.

### MSC manufacture and administration

MSCs were derived from UCs of unrelated HLA-mismatched donors. The culture and expansion of UC-MSCs were carried out by modifying methods previously published (21, 22). Briefly, UC tissues were digested with 0.05% type II collagenase (Sigma, St Louis, USA), and the cell suspension was collected by filtering through a stainless-steel mesh. The cells were resuspended in serum-free MSC culture media. After culturing, non-adherent cells were discarded. The adherent cells were detached using 0.05% Trypsin and 0.01% EDTA (Gibco, Grand Island, NY, USA). The fifth-passage cells were frozen. Each batch of UC-MSCs was characterized by flow cytometry for phenotype and, in some cases, tested for their ability to differentiate into adipocytes, osteoblasts, and chondrocytes. The UC-MSCs suspensions were cultured and tested negative for bacteria and mycoplasma contamination before infusion.

UC-MSCs therapy was initiated as soon as possible after the onset of steroid refractory grade III-IV acute GVHD. Patients received intravenous infusions of cryopreserved and freshly thawed MSCs at a dose of 1×106/kg, either once or twice a week, depending on their symptom severity. These infusions were given in conjunction with corticosteroids and cyclosporine until aGVHD showed a response.

### Evaluation points

The primary endpoint of the study was to assess the efficacy of UC-MSCs therapy, which was evaluated based on complete response (CR), partial response (PR), and overall response (OR) rates. CR was defined as the complete resolution of all symptoms of aGVHD. PR was defined as a clinical improvement of at least one GVHD grade. OR encompassed both CR and PR. Response to UC-MSCs therapy was evaluated on day 28, day 56 and day 100 after the first MSC infusion, or on the date of death if it occurred before 28 days. Patients who showed no change in their disease status (stable disease, SD) or those who experienced worsening symptoms (progressive disease, PD) were classified as having no response (NR).

### Statistical analysis

Response rates across different categories were compared using Fisher’s exact test. This test allowed us to determine whether there were significant differences in response rated between different groups. To estimate the probability of survival, we used the Kaplan–Meier method. This method allows us to estimate the survival function from the observed data. We compare survival curves between groups using the log-rank test. A P-value of less than 0.05 was considered statistically significant, indicating that the observed differences in response rated or survival probabilities. All Statistical analyses were performed using the statistical software R.

## Results

### Patient characteristics

Between September 2010 and April 2018, a total of 86 patients were enrolled in this study. Patient characteristics are summarized in **Table 1**. The median age of these patients was 27.5 years old (ranging from 11 to 54 years). The majority of patients, 37 in total, received allogeneic HSCT due to acute myeloid leukemia (AML). Other indications for HSCT included acute lymphoblastic leukemia (ALL) in 27 patients, myelodysplastic syndrome (MDS) in 8, chronic myeloid leukemia (CML) in 4, aplastic anemia (AA) in 3, non-Hodgkin lymphoma (NHL) in 2, and other diseases in 5 patients. HSCT was performed using granulocyte colony-stimulating factor (G-CSF) mobilized peripheral blood stem cells in 84 patients, BM in 1 patient, and a combination of both in another patient. Donors were either HLA-compatible in 19 cases or partially HLA matched in 67 cases. Myeloablative regimens were used in all cases. Forty-four patients received ATG as part of their conditioning regimen. GVHD prophylaxis consisted primarily of CsA combined with methotrexate and MMF in 44 patients. All patients developed lower GI tract aGVHD while receiving prophylactic immunosuppressive drugs. The median time from HSCT to the onset of lower GI tract aGVHD was 37 days (ranging from 7 to 216 days). The majority of patients, 71 out of 86 (82.6%), presented with grade IV acute lower GI tract GVHD. Additionally, 18 patients (20.9%) developed both acute lower GI tract and liver GVHD. None of the patients had used ruxolitinib.

**Table 1:** Characteristics of patients.

**Sex, n(%)**			
	Male	57	(66)
	Female	29	(34)
**Age at HSCT, n(%)**	**Median: 27.5 (11-54)**	** **	** **
	<18 y	17	(20)
	18-25 y	21	(24)
	>25 y	48	(56)
**Primary disease, n(%)**			
	AML	37	(43)
	ALL	27	(31)
	MDS	8	(9)
	CML	4	(5)
	AA	3	(3)
	NHL	2	(2)
	Others	5	(6)
**Disease status at HSCT, n(%)**			
	CR	62	(72)
	PR	19	(22)
	NR	5	(6)
**Type of donor, n(%)**			
	MUD	52	(60)
	MSD	20	(23)
	Haploidentical	14	(16)
**HLA typing, n(%)**			
	HLA-identical	19	(22)
	9/10	21	(24)
	8/10	20	(23)
	7/10	7	(8)
	6/10	2	(2)
	5/10	15	(17)
	other	2	(2)
**Hematopoietic stem cell source, n(%)**			
	PBSC	84	(98)
	BM	1	(1)
	PBSC + BM	1	(1)
**GVHD Prophylaxis, n(%)**			
	CSA+MTX+MMF	44	(51)
	CSA+MTX+MMF+MVC	7	(8)
	CSA+MTX	33	(38)
	CSA+MTX+MVC	2	(2)
**Onset of aGVHD (days)**	**Median: 37 (7-216)**	** **	** **
**Gut aGVHD grade, n(%)**			
	III	15	(17)
	IV	71	(83)
**with liver aGVHD, n(%)**			
	Yes	18	(21)
	No	67	(78)
	NR	1	(1)

AML, acute myeloid leukemia; ALL, acute lymphoblastic leukemia; MDS, myelodysplastic syndrome; CML, chronic myelogenous leukemia; AA, aplastic anemia; NHL, non-Hodgkin lymphoma; CR, complete response; PR, partial response; NR, no response; MUD, matched unrelated donor; MSD, matched sibling donor; PBSC, peripheral blood stem cell; BM, bone marrow; CsA, cyclosporine A; MTX, methotrexate; MMF, mycophenolate mofetil; MVC, maraviroc; ATG, antithymocyte globulin.

### Treatments of aGVHD before MSCs infusions

All patients enrolled in the study received steroids as first-line treatment for aGVHD, but none responded to this therapy. Subsequently, 30 patients (34.9%) received one or two second-line immunosuppressive drug (these patients were treated with either a CNI alone or in combination with Basiliximab), while 56 patients (65.1%) did not respond to three or more additional immunosuppressive therapies. The median time from the diagnosis of aGVHD to the first infusion of UC-MSCs was 7 days (ranging from 3 to 88 days). The cell dose per infusion was 1 x 106/kg. Each patient received a median of 4 infusions, with a range of 1 to 16 infusions. The UC-MSCs therapy was well-tolerated, and no acute or late side effects were observed during or after the infusions.

### Response to MSC treatment

**Figure 1:**
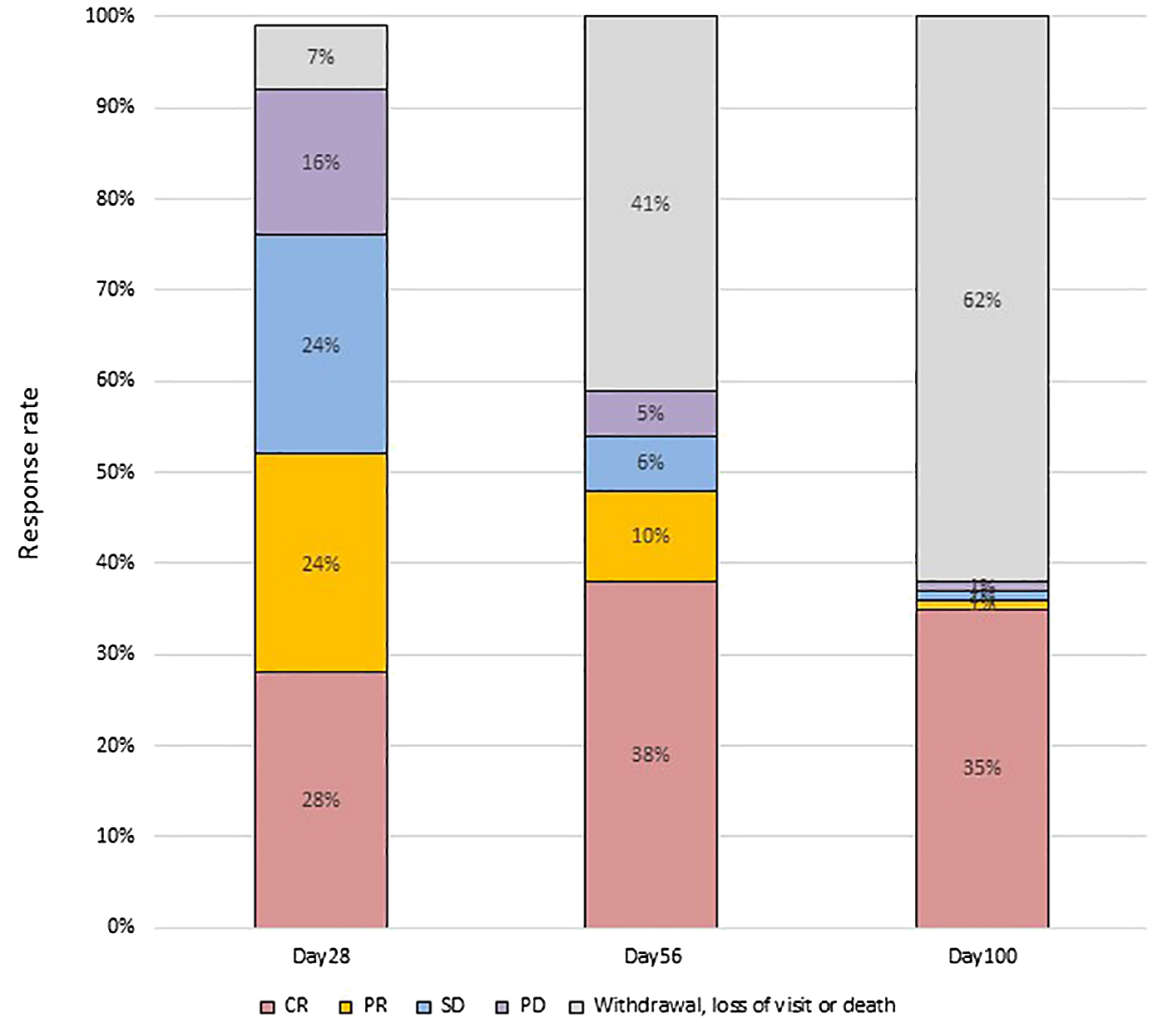
GVHD response and outcome.

**Figure 2:**
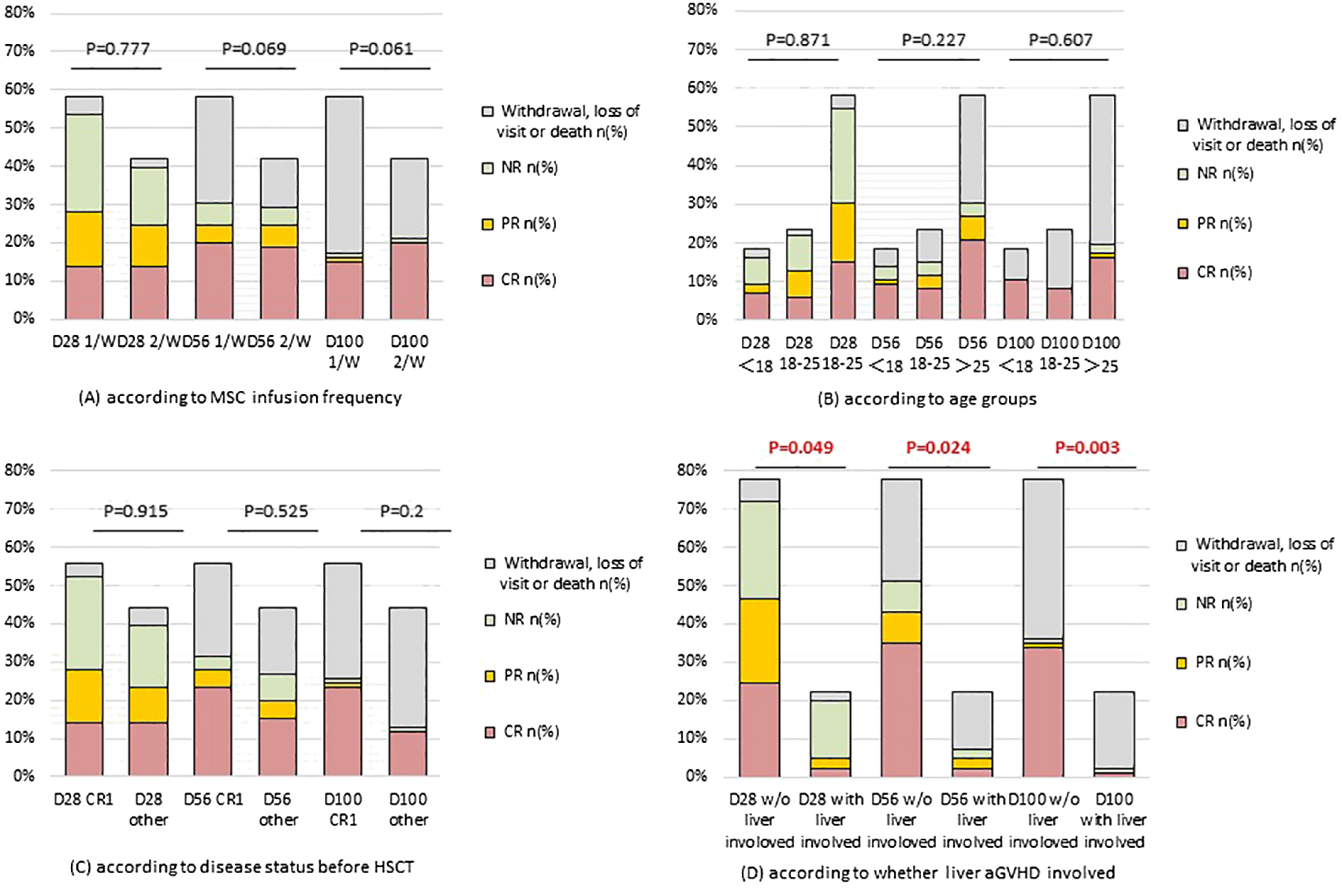
GVHD response in different subgroups. **(A)** according to MSC infusion frequency; **(B)** according to age groups; **(C)** according to disease status before HSCT; **(D)** according to whether liver aGVHD involved. D28/56/100: 28/56/100 days after the first infusion of UC-MSCs. W/O, without. NR, not response. PR, partial response. CR, complete response.

At day 28 post-treatment, over half of the patients (45/86, 52.3%) achieved an OR, including 24 patients (27.9%) who achieved a CR and 21 (24.4%) who achieved a PR (**Figure 1**). Forty-three patients did not respond to the treatment, with 21 (24.4%) showing SD, 14 (16.3%) showing PD, and 6 (7.0%) dying. Patients who developed both acute lower GI tract and liver GVHD had significantly worse OR at day 28 compared to patients who only had acute lower GI tract GVHD (22.2% vs. 58.8%; p= 0.049) (**Figure 2**).

There were no significant differences in clinical responsiveness based on age groups. Among children under 18 years old, 6 (37.5%) reached CR and 2 (12.5%) achieved PR on day 28. Among patients aged 18-25 years, 5 (25.0%) achieved CR, 6 (30.0%) achieved PR. Among patients above 25 years of age, 13 (26.0%) achieved CR, 13 (26.0%) PR. Similarly, there were no significant differences in OR between patients receiving once or twice weekly infusions of UC-MSCs (48.0% vs. 58.3%; p= 0.777). Furthermore, there were no differences in OR based on whether patients were in CR1 status before transplantation (50.0% vs. 54.1%, p=0.915) (**Figure 2**).

### Survival

**Figure 3:**
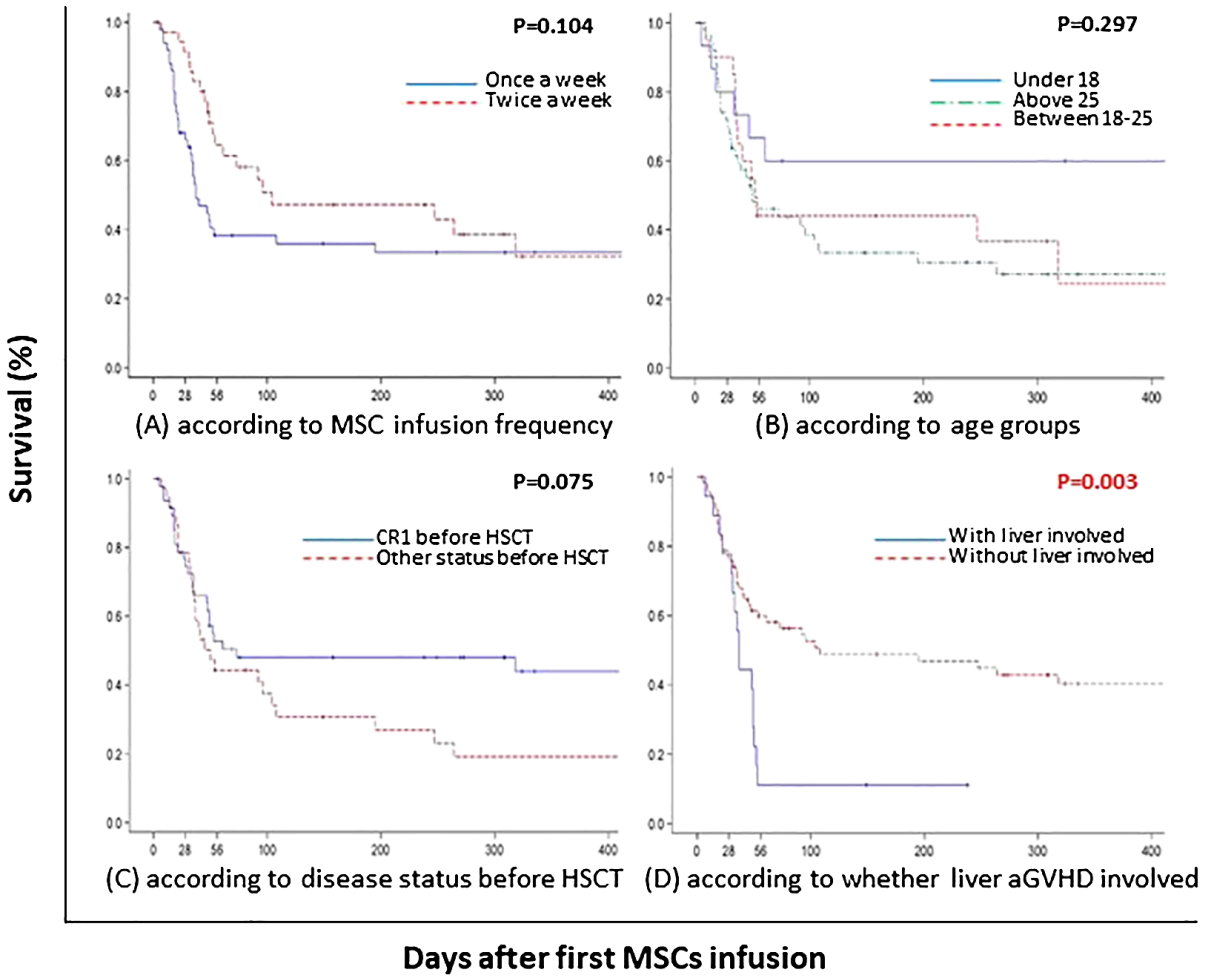
One-year survival estimates for patients with steroid-resistant and therapy-refractory III-IV acute GI GVHD from time of first MSC infusion. **(A)** according to MSC infusion frequency; **(B)** according to age groups; **(C)** according to disease status before HSCT; **(D)** according to whether liver aGVHD involved. Abbreviations: aGVHD, acute graft-versus-host disease; HSCT, hematopoietic stem cell transplantation; CR, complete response; MSC, mesenchymal stem cell.

The overall survival (OS) at 100 days was 43.7% for the entire cohort of patients, and 60.0% for children specifically (P=0.297). When stratified by GVHD involvement, the OS was significantly higher for patients with only acute lower GI tract GVHD (52.6%) compared to those with liver involvement (11.1%) (P=0.003) (**Figure 3**).

As of the last follow-up in December 2023, 10 patients (11.6%) were alive, with a median follow-up duration of 108 months (ranging from 61 to 159 months) from the first infusion of UC-MSCs. Notably, none of the survivors experienced recurrence of their original disease or development of secondary tumors, leading to a DFS rate of 11.6%. Ten patients were out of touch and 66 had passed away: 17 due to infections, 8 due to disease relapses, 22 due to the progression of GVHD, 2 due to TMA, 1 due to bleeding, and for 16, the cause of death was indeterminate.

## Discussion

The present study represents the largest single-center cohort of 86 patients treated with UC-MSCs for steroid refractory acute GVHD. It suggests the use of umbilical cord as a source of MSCs seems to have similar results with that of bone marrow. In our study, an OR of 52.3% (45 of 86 patients) was observed by day 28 following UC-MSCs infusion. This is comparable to a previous single-center study reporting an OR of 59% in 54 children treated with UC-MSCs for grades II-IV aGVHD (15). And our study indicates that UC-MSCs maintain its safety profile even after long-term follow-up.

Currently, BM-MSCs have been approved in Japan, Canada and New Zealand for the treatment of GVHD. Although clinical studies of MSCs for aGVHD have generally shown encouraging efficacy results, the response rates can vary. Many factors are likely to influence the outcomes: expansion protocols, MSC dose per infusion, number of infusions, patient age, and choice of second-line agents. The recent review concludes several trials with 30 or more patients treated with BM-MSC, the OS by day 28 ranging from 40% to 82.8% (23). In the field of UC-MSC therapy, Ding Y, et al. studied 54 patients with grade II-IV aGVHD, with the majority (74%) at grade III and 14.8% at grade IV. The median age was 12.5 years, spanning from 1 to 62 years old. The 28-day OR rate was 59.3% (15). Donadel CD, et al. presented data on 52 patients with grade II-IV aGVHD, with 25% at grade III and 71.2% at grade IV. The median age was also 12.5 years, but the age range was broader, from 0.3 to 65 years old. Their 28-day OR rate was slightly higher at 63.5% (16). Zhao, et al. reported on a less severe cohort of 25 patients with aGVHD of grade II-IV, with 44% having grade III/IV. This group had a higher median age of 37 years, ranging from 24.5 to 47 years old. They achieved a notably higher 28-day OR rate of 80% (18). The relatively inferior results reported in our study could be attributed to the severity of the disease in our patient cohort. Most (82.6%) of our patients had grade IV steroid refractory aGVHD, which is much more severe than most published series (grade II to IV). Our study’s emphasis is on GI aGVHD, with or without liver involvement. This is because skin-limited aGVHD (stage 4 limited-skin aGVHD are also categorized as grade IV) typically responds well to steroid therapy and is less likely to be life-threatening (24, 25). Additionally, most of our patients had received more than two additional treatments (none of the patients ever received ruxolitinib) before UC-MSCs infusion, indicating that they were receiving MSCs as a salvage therapy. It has suggested that patients with severe lymphodepletion due to GVHD and multiple immunosuppressive treatment regimens may lose responsiveness to MSCs (23). Despite these challenges, the results we obtained from such a highly challenging patient cohort (17.4% grade III and 82.6% IV) suggest the advantage of treatment of aGVHD with UC-MSCs.

The association between liver involvement and a worse response, as observed in our study, is consistent with other reports (15, 26, 27). This suggests that liver involvement is a prognostic factor for aGVHD, rather than a specific predictor for MSC therapy. However, it is noteworthy that a prospective randomized trial found that remestemcel-L led to significantly higher overall response rates in GVHD patients with liver involvement (28). This finding highlights that MSCs cannot be excluded from the treatment options for liver aGVHD patients. Further exploration of MSCs as a treatment option for aGVHD patient with liver involvement is needed.

Our study’s observation of similar response rates between children and adults (P = 0.871) is consistent with recent reports on UCB-MSCs (15) and a study using Temcell (27). This suggests that MSCs may have similar efficacy in both pediatric and adult patients with aGVHD. However, this finding disagrees with most studies that have demonstrated a trend towards a better clinical response in children both in an UC-MSC report (16) or in BM-MSC reports (28–31). This discrepancy could be due to differences in patient populations, disease severity, or other confounding factors.

Regarding the frequency of MSC infusion, our study did not find a significant difference in response rates between once-weekly or twice-weekly infusions. However, Larger controlled studies are needed to definitively identify the optimal infusion schedule for MSCs in the treatment of aGVHD.

In our study, the administration of UC-MSCs did not elicit any adverse effects among patients, thus confirming its safety. As of the last follow-up in December 2023, our cohort, with a median follow-up of 108 months (ranging from 61 to 159 months) from the initial UC-MSCs infusion, exhibited a survival rate of approximately 11.6% (10/86).

We noticed that a phase II study showed a pre-MSC six-biomarker (IL2Rα, TNFR1, HGF, IL-8, Elafin and Reg3α) panel and post-MSC ST2 levels were predictive of mortality (32). However, over the past few years, we have not made it a standard practice to monitor the cell subsets or plasma aGVHD biomarkers. In the future study, we will initiate the monitoring of biomarkers before and after administration of UC-MSCs therapy.

UC-MSCs offer a promising therapeutic potential due to their non-invasive acquisition and comparable clinical efficacy to bone marrow-derived stem cells. This study provides important insights into the clinical outcomes and safety profile of UC-MSC therapy in the treatment of aGVHD, particularly in patients with liver involvement and in both pediatric and adult populations. Future randomized, placebo-controlled, and double-blind studies are necessary to identify the most suitable patient populations, predictors of response, optimizing infusion schedules, and exploring combinations of MSCs with other immunosuppressive agents to further improve outcomes for patients with aGVHD.

## Data Availability

The datasets presented in this article are not readily available because only the editorial office could apply to the dataset during the review process. Requests to access the datasets should be directed to LH: huliangding@sohu.com.
